# IL-2 and Anti-TGF-β Promote NK Cell Reconstitution and Anti-tumor Effects after Syngeneic Hematopoietic Stem Cell Transplantation

**DOI:** 10.3390/cancers12113189

**Published:** 2020-10-29

**Authors:** Maite Alvarez, Cordelia Dunai, Lam T. Khuat, Ethan G. Aguilar, Isabel Barao, William J. Murphy

**Affiliations:** 1 Department of Dermatology, University of California, Davis, Sacramento, CA 95817, USA; malvarezr@unav.es (M.A.); cdunai@ucdavis.edu (C.D.); ltkhuat@ucdavis.edu (L.T.K.); eaguilar@umn.edu (E.G.A.); isilvestredegals@ucdavis.edu (I.B.); 2 Program for Immunology and Immunotherapy Department, Center for Applied Medical research (CIMA), Universidad de Navarra, 31008 Pamplona, Spain; 3 Navarra Institute for Health Research (IdiSNA), 31008 Pamplona, Spain; 4 Centro de Investigación Biomédica en Red de Cáncer (CIBERONC), 28029 Madrid, Spain; 5 Department of Pediatrics, Division of Blood and Marrow Transplantation, University of Minnesota, Minneapolis, MN 55455, USA; 6 Department of Internal Medicine, University of California, Davis, Sacramento, CA 95817, USA

**Keywords:** HSCT, IL-2, TGF-β, NK cells, myeloid cells, immune reconstitution

## Abstract

**Simple Summary:**

Hematopoietic stem cell transplantation (HSCT) causes early immune deficiency and susceptibility to both opportunistic infections and cancer relapse. In this study, using a mouse model where donor cells can be tracked over time, we have observed that the combination of IL-2 (a cytokine which activates the immune system) combined with the blockade of TGF-β (a cytokine which suppresses the immune system) increased immune recovery and resulted in greater anti-tumor efficacy. The combination of IL-2 and anti-TGF-β accelerated NK cell and myeloid cell reconstitution after HSCT.

**Abstract:**

The failure of autologous hematopoietic stem cell transplantation (HSCT) has been associated with a profound immunodeficiency that follows shortly after treatment, which renders patients susceptible to opportunistic infections and/or cancer relapse. Thus, given the additional immunosuppressive pathways involved in immune evasion in cancer, strategies that induce a faster reconstitution of key immune effector cells are needed. Natural killer (NK) cells mediate potent anti-tumor effector functions and are the first immune cells to repopulate after HSCT. TGF-β is a potent immunosuppressive cytokine that can impede both the development and function of immune cells. Here, we evaluated the use of an immunotherapeutic regimen that combines low dose of IL-2, an NK cell stimulatory signal, with TGF-β neutralization, in order to accelerate NK cell reconstitution following congenic HSCT in mice by providing stimulatory signals yet also abrogating inhibitory ones. This therapy led to a marked expansion of NK cells and accelerated NK cell maturation. Following HSCT, mature NK cells from the treated recipients displayed an activated phenotype and enhanced anti-tumor responses both in vitro and in vivo. No overt toxicities or adverse effects were observed in the treated recipients. However, these stimulatory effects on NK cell recovery were predicated upon continuous treatment as cessation of treatment led to return to baseline levels and to no improvement of overall immune recovery when assessed at later time-points, indicating strict regulatory control of the NK cell compartment. Overall, this study still demonstrates that therapies that combine positive and negative signals can be plausible strategies to accelerate NK cell reconstitution following HSCT and augment anti-tumor efficacy.

## 1. Introduction

Hematopoietic stem cell transplantation (HSCT), both autologous or allogeneic is used to treat a variety of hematological malignancies, such as leukemia or lymphoma [1]. Despite the benefits that this therapy has shown with hematologic cancers, there still are significant obstacles associated with it limiting efficacy including opportunistic infections such as CMV reactivation, cancer relapse and susceptibility to opportunistic infections due to a profound state of immune deficiency following the transplant. In addition, with allogeneic HSCT, the occurrence of graft-versus-host disease (GvHD), which accounts for a significant degree of the HSCT-related mortality and morbidity necessitating immunosuppression [1,2]. Given these shortcomings associated with HSCT, the development of means to accelerate immune reconstitution post-transplant may significantly impact outcomes, particularly with relapse.

Natural killer (NK) cells have demonstrated to be the first lymphoid cell to repopulate the immune system following HSCT [2,3]. As members of the innate immune system, NK cells have the ability to eliminate target cells without prior immunization and are known to have potent anti-viral and anti-tumor effects, particularly against leukemia and metastatic tumors. Several NK cell-based immunotherapies have been administered in combination with HSCT with some success [4]. However, despite significant progress in NK cell expansion ex vivo, clinical benefits from NK cell adoptive transfer therapy has been rather limited [4,5], possibly due to suppressor mechanisms, issues with the short life-span of NK cells, lack of optimization of NK cell transfer conditions or immune evasion. Therefore, new strategies to improve NK cell development and function after HSCT are necessary particularly since T cell reconstitution occurs markedly later after HSCT.

NK cells are highly regulated by the presence of inhibitory and activating receptors on their surfaces that prevent NK activation in the presence of self-ligands as well as to recognize possible stress signals on target cells [6,7,8]. In the mouse, many of these inhibitory and activating receptors belong to the Ly49 and natural cytotoxicity receptor (NCR) families [9]. NK development and activation is also regulated by the presence of many cytokines such as interleukin(IL)-15 [10,11] type I interferons (IFN), IL-12 and IL-18 [12]. IL-2 is another cytokine involved in NK cell activation and has been frequently used to expand NK cells both in vivo and in vitro [12]. However, the high and frequent doses necessary to achieve anti-tumor efficacy have limited its therapeutic use due to marked toxicities as well as potential effects on expanding Tregs [1]. In particular, the toxicities associated with systemic IL-2 administration is why efforts have been focused on combinatorial therapies in order to reduce toxicities and increase efficacy. Studies in mouse models have shown successful anti-tumor effects when IL-2 was combined with regulatory T cell (Treg) depletion by monoclonal CD25 antibody [13]. Other approaches are looking to enhance the IL-2 stability that increase the half-life of this cytokine allowing for reductions in the frequency and doses required. The use of a particular clone of anti-IL-2 (S4B6.1) has been shown to stabilize IL-2 and induce CD8 T cell and NK cell expansion while not affecting Tregs [14].

NK cell function and expansion can be inhibited by the suppressor molecule tumor growth factor-β (TGF-β). TGF-β is involved in cell proliferation, differentiation, migration and survival of multiple cell-types, particularly hematopoietic-derived cells. It has pleiotropic effects including fibrosis, wound healing, hematopoietic cell differentiation and expansion as well as on immune responses [15]. Although it has been reported that Tregs secrete TGF-β to regulate the immune response upon activation, TGF-β is produced by most of the cells in the body. TGF-β can also be produced by multiple tumor types in order to evade the immune response as well as promote progression [16,17]. TGF-β is a potent inhibitor of NK cell function through attenuation of cytolytic activity and IFN-γ production [18,19]. Moreover, it can induce downregulation of activating receptors such as Natural killer group 2, member D (NKG2D) resulting in poor tumor lysis capabilities [20]. TGF-β can also impact the reconstitution of myeloid cells after HSCT. The neutralization of TGF-β has been looked at as a cancer therapy for preventing tumor-sensitized Tregs [16,17,21], augmenting anti-tumor responses [22] and inhibiting metastases [15,23]. TGF-β blockade was also shown to restore NKG2D levels as well as cytolytic functions [24]. The suppression of TGF-β signaling also results in an effective expansion of myeloid-derived cells [25]. Currently, there are several phase I/III clinical trials using antisense TGF-β oligonucleotides that inhibit TGFβ-1 (AP 12009) in patients with recurrent high-grade glioma. The use of a mAb that neutralizes the three isoforms of TGF-β (clone 1D11) in combination with a vaccine targeting glioma-associated antigen-derived CD8 T cells resulted in synergistic expansion of antigen-specific T cells and prolonged survival of tumor-bearing mice [26]. Recently, we and others have demonstrated that combination therapy (CT) with co-administration of IL-2 and TGF-β neutralization in tumor-bearing mice results in the expansion of NK and CD8 T cells and consequently enhanced anti-tumor responses [27,28]. In this study, we sought to evaluate the impact of this CT after HSCT to determine effects on NK cell recovery. We observed that this CT regimen given early after HSCT resulted in accelerated NK cell reconstitution and enhanced function resulting in greater anti-tumor effects with no toxicities suggesting this approach may be of use in HSCT.

## 2. Material and Methods

### 2.1. Mice

Female C57BL/6 (CD45.2) were purchased from Taconic Farm Inc. (Hudson, NY, USA). Female congenic Ly5.1 (CD45.1) were purchased from the Animal Production Area, National Cancer Institute (Frederick, MD, USA). All mice were used at 8–12 weeks of age and housed under specific pathogen-free conditions in the Animal Facility of the University of California, Davis. The University of California, Davis Institutional Animal Care and Use Committee (IACUC) approved studies and protocols (Protocol# 18940).

### 2.2. Hematopoietic Stem Cell Transplantation

C57BL/6 (CD45.2) mice were conditioned with a lethal dose of 950 cGy of gamma-irradiation from a Cesium (Cs) source, while BALB/c received 800 cGy. Donor Ly5.1 (CD45.1) or syngeneic bone marrow (BM) cells were obtained after flushing femurs, tibias and backbones under aseptic conditions. One to five million BM cells were infused intravenously (iv) into the recipient irradiated mice (specified in figures). At day 11 post-HSCT the animals received daily intraperitoneal (ip) injections of 0.2–1 × 10^6^ international unit (IU) of recombinant human IL-2 (rhIL-2, National Cancer Institute repository) and/or 240 µg of anti-TGF-β (clone 1D11) every other day for a week. Control groups received phosphate buffered saline (PBS) and/or Rat-IgG (rIgG, Jackson Immunoresearch, West Grove, PA, USA) when appropriate. Spleen and BM were harvested on day 18 and 25 post-HSCT. Each group consisted of 3–4 animals.

### 2.3. Flow Cytometry

The following antibodies (Ab) were purchased from Becton Dickinson (BD) Biosciences (San Jose, CA, USA): FITC anti-Ly49G2 (4D11), FITC anti-CD4 (GK1.5), FITC anti-Ly6C/G (Gr1), FITC and PE anti-CD8 (53-6.7), PE anti-Ly49C/I (5E6), PE anti-CD3 (17A2), PE anti-CD19 (1D3), PE and APCCy7 anti-CD11b (M1/70) and APCCy7 anti-CD25 (PC61); from eBioscience (San Diego, CA, USA): FITC anti-F4/80 (BM8), FITC anti-CD27 (L6.79), PE anti-NKG2A (16A11), PECy5 anti-CD4, PECy5 anti-CD62L (MEL-14), PECy7 anti-NKG2D (IM7) and biotin anti-Ly49D (AE5); from BioLegend (San Diego, CA, USA): PB anti-CD45 (30-F11), PB anti-CD45.1 (A20), PB anti-Thy1.2 (30-H12), PB anti-CD44 (IM7), PECy7 anti-CD3 (145-2C11), PECy7 CD11c (N418), alexafluor^®^ (AF) 647 anti-NK1.1 (PK136), AF700 anti-CD8, APC anti-CD49b (clone DX5), biotin anti-CD122 (5H4) and APC- or PerCPCy5.5-conjugated streptavidin. Single-cell suspensions were prepared and antibody staining was performed as previously described [29,30]. Stained cells were analyzed with an LSR-Fortessa cytometer (Becton Dickinson) and FlowJo software (TreeStar, Ashland, OR, USA) was used for data analysis.

For intracellular staining, PE anti-IFN-γ from BD or PE anti-Granzyme B from Invitrogen (Grand Island, NY, USA) were used. For regulatory T cell detection FITC anti-Foxp3 (FJK-16a) and Foxp3 intracellular staining kit from eBioscience were used following manufacturer’s instructions. Isotype-matched mouse and rat IgG mAbs were used as negative staining controls. Nonspecific binding was prevented using the FcγII and FcγIII receptor anti-CD32/CD16 (2.4G2) mAb from eBioscience (San Diego, CA, USA).

### 2.4. Cytotoxic Assays

A standard 4-hour ^51^Cr-release assay was performed to determine NK cell lytic function [28]. Briefly, splenocytes of treated mice were cultured with ^51^Cr-labeled tumor cells from the NK-sensitive tumor cell line YAC-1 (ATCC: Manassas, VA, USA) at different effector: target ratio (E:T) during 4-hours and then supernatants were collected to determine the amount of ^51^Cr released from dead tumor cells using a LKB-gamma counter (Pharmacia, Uppsala, Sweden). Percentage of tumor lysis was calculated as previously described [29].

### 2.5. Serum Cytokine Bead Array

Serum cytokine levels of TNF-α and IFN-γ, were quantified using the Becton Dickinson Cytometric Bead Array (CBA) (BD Biosciences) kit for flow cytometric assay following manufacturer’s instructions.

### 2.6. In Vivo Tumor Models

C57BL/6 mice were iv injected with 10^5^ mouse Lewis lung carcinoma (3LL) tumor cells or mouse acute myeloid leukemia cell line (C1498, ATCC) four days prior to HSCT or one day after HSCT respectively. Mice were treated with IL-2/ anti-TGF-β (1D11) following the regimen described in the previous section starting on day 11 post-HSCT. Anti-NK1.1 or rIgG was given when appropriate two days before starting the CT regimen and once a week until the end of the experiment to deplete the NK cell population. BALB/c female mice were iv injected with 2 × 10^5^ luciferase+ A20 cells at day 6 post syngeneic HSCT. Mice were administered with anti-asialo-GM1 (anti-ASGM1, Wako Chemicals, Richmond, VA, USA) or isotype control intraperitoneally every four days starting on day 4. Mice were treated with IL-2/ anti-TGF-β (1D11) following the regimen described in the previous section starting on day 11 post-HSCT. Mice were then monitored for survival and imaged for bioluminescense (A20 studies, IVIS Spectrum in vivo imaging system, Perkin Elmer, Waltham, MA, USA) and mice were euthanized at humane endpoints in accordance with UC Davis IACUC guidelines.

### 2.7. Statistical Analysis

Each experiment was performed at least two times with 3–4 mice per group. Student’s two-tailed *t*-test, one-way ANOVA (Tukey post-test analysis), two-way ANOVA (Bonferroni post-test analysis) or Log-rank test were used when appropriate to determine statistical significance. *P* values were considered statistically significant when *p* < 0.05.

## 3. Results

### 3.1. IL-2 and Anti-TGF-β Combination Therapy (CT) Results in Marked NK Cell Expansion after Congenic HSCT

**Figure 1:**
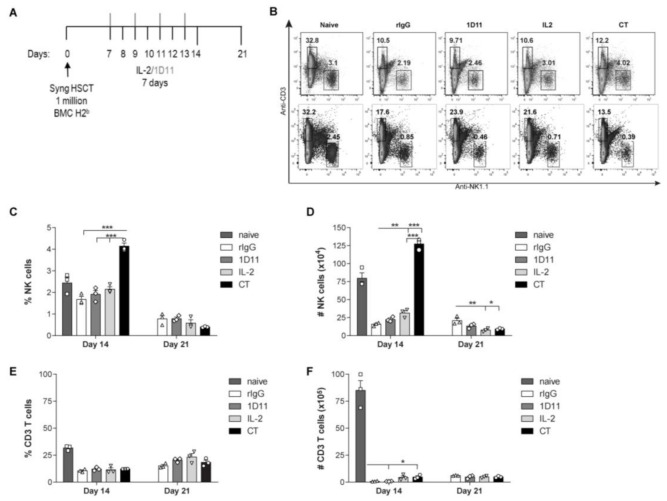
IL-2 and anti-TGF-β treatment shortly after HSCT induces a transitory but strong NK cell expansion. Spleens from treated C57BL/6 mice after HSCT were harvested 24 hours (14 days post-HSCT) or a week (21 days post-HSCT) after end on treatment and NK cells were analyzed by flow cytometry. (**A**) Schematic representation dose regimen is shown. (**B**) Representative dot plots of gated NK cells (CD3^−^NK1.1^+^) or T cells (CD3^+^NK1.1^−^) at day 14 (upper panel) and 21 (lower panel) post-HSCT are shown. (**C**,**D**) Percentage and total number of NK cells are shown at day 14 and day 21 after HSCT for gated CD3^−^NK1.1^+^. (**E**,**F**) Percentage and total number of CD3 T cells are shown at day 14 and day 21 after HSCT for gated CD3^+^NK1.1^−^. The percentage and numbers of NK and CD3 T cells from naïve no treated mice are shown for comparison. Data are representative of at least two independent experiments with three mice per group (mean ± SEM). One-Way ANOVA was used to assess significance (* *p* < 0.05, ** *p* < 0.01, *** *p* < 0.001).

We have previously demonstrated that administration of this CT regimen in resting mice lead to a significant increase of NK cells in multiple organs and was also accompanied by improved NK cell activity and function evidenced by prolonged survival in tumor-bearing mice [28]. To improve the clinical relevance of this therapy and given the role of NK cells in early protection after HSCT, we hypothesized that application of IL-2 and anti-TGF-β therapy after HSCT would improve NK cell reconstitution. C57BL/6 mice (CD45.2^+^) received 10^6^ CD45.1^+^ Ly5.1 congenic BMCs after lethal radiation. Because NK cell recovery after HSCT has been shown to begin around day 7 post-HSCT, we initiated immunotherapy at this time to ensure the benefits of the therapy on NK cells as other immune cells present at earlier time points post-HSCT could be expanded by IL-2 as well. Mice were treated daily for 7 days with 2 × 10^5^ IU of IL-2 and/or 240 μg of anti-TGF-β every other day and organs were collected 24 h (day 14 post-HSCT) and 7 days (day 21 post-HSCT) after the end of IL-2/anti-TGF-β treatment (Figure 1A).

Flow cytometry analysis revealed that CT resulted in a significant expansion of both the percentage and total numbers of NK cells at day 14 post-HSCT compared to IL-2 treatment alone demonstrating an additive effect of anti-TGF-β (Figure 1B–D). However, consistent with what was observed in resting mice [28], the effect on NK cells was not present a week after cessation of treatment (21 days post-HSCT) (Figure 1B–D). This temporary effect was consistent with the short half-life of both IL-2 and anti-TGF-β [31,32] as well as the result of the already described addictive or contraction effect that leads to the loss of NK cells after the cessation of IL-2 treatment [33]. CT treatment for 7 days resulted in a better impact on NK cell expansion compared to 3 days treatment that ensured an improvement on the NK cell compartment in correlation with naive mice (Figure S1). This is important because faster recovery of immune cells at the steady state levels is a desirable outcome for HSCT therapy. Additionally, the contribution of anti-TGF-β treatment was likely due to the blockade of the immunosuppressive effect of Tregs, as a significant expansion of Tregs was detected after IL-2 treatment at day 7 post-HSCT (Figure S2). It is also important to note that a higher dose of IL-2 diluted the anti-TGF-β additive effect because no differences in NK cell expansion were observed between anti-TGF-β treatment combined with low dose of IL-2 (LD: 2 × 10^5^ IU) or intermediate dose (ID: 5 × 10^5^ IU) and the effect was equivalent to that of high dose IL-2 (HD: 10^6^ IU) (Figure S1). Importantly, the immunotherapy was well tolerated, as mice did not display any signs of distress associated with high-dose IL-2 toxicity. 

In contrast, the impact of CT immunotherapy on the T cell compartment was negligible, evidenced by the relatively low percentages and total numbers of CD3 T cells when compared to naïve mice (Figure 1B,E,F). These results were expected because the reconstitution of T cells occurs at later time points after congenic HSCT (greater than 35 days) and CT therapy was primarily aimed to influence the NK cell expansion and reconstitution which occur early (day 7) after HSCT and represents a critical period for relapse potential. These results suggest that IL-2 and anti-TGF-β cooperate in an additive manner for the induction of a transient but stronger NK cell expansion compared to IL-2 alone.

### 3.2. Administration of IL-2 Combined with Anti-TGF-β Accelerates NK Cell Reconstitution after HSCT

**Figure 2:**
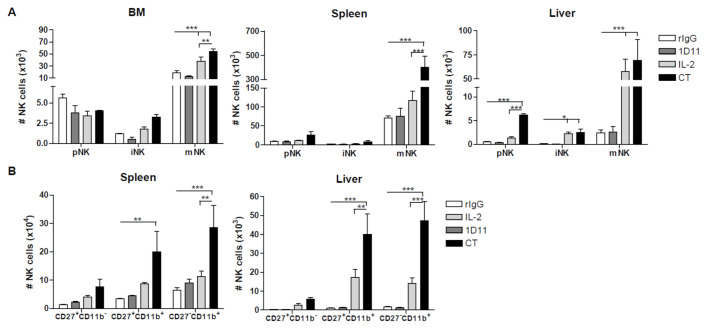
NK cell stimulation after HSCT results in accelerated reconstitution enhanced by TGF-β blockade. Cells from spleen, liver and BM were stained to determine the different NK cell developmental stages defined by precursor (pNK: CD45.1^+^CD3^−^CD122^+^NKG2D^+^NK1.1^−^DX5^−^), immature (iNK: CD45.1^+^CD3^−^CD122^+^NKG2D^+^NK1.1^+^DX5^−^) and mature (mNK: CD45.1^+^CD3^−^CD122^+^NKG2D^+^NK1.1^+^DX5^+^) NK cells. (**A**) Total number of pNK, iNK and mNK cells is shown for BM, spleen and liver at day 14 post-HSCT. Spleen and liver cells were stained for CD27 and CD11b to further differentiate mature properties. (**B**) Total number of mNK cells differentiated in function of CD27 and/or CD11b expression is shown. Data are representative of at least two independent experiments with three mice per group (mean ± SEM). One-Way ANOVA was used to assess significance (* *p* < 0.05, ** *p* < 0.01, *** *p* < 0.001).

The negative impact of TGF-β in NK cell ontogeny has been previously described [34]. In agreement with this study, we also observed that the administration of CT in resting mice resulted in the recruitment of mature NK cells from progenitor cells [28]. We hypothesized that NK cell stimulation with IL-2 combined with TGF-β neutralization would not only induce an expansion of mature NK cells but also a faster NK cell maturation from progenitors present in the BM after HSCT thereby improving overall NK cell reconstitution. Analysis of NK cell maturation stages [28,34] revealed that while the majority of NK cells were phenotypically mature (mNK) after NK cell stimulation, an elevated proportion of NK cells were present in the precursor stage (pNK) in spleen and liver and the immature stage (iNK) in BM, spleen and liver compared to rIgG (Figure 2A). Therefore, accelerated NK cell reconstitution can be implied by the fact that the total number of mNK cells is significantly higher than pNK and iNK cell in NK cell developmental organs after CT treatment. Further analysis of the mature NK cell compartment revealed the presence of NK cells with intermediate, mature-like properties [35,36] due to the high proportion of mNK cells that co-expressed CD11b and CD27, which is similar to the more mature phenotype CD11b^+^CD27b^−^ NK cell subset also suggesting a continuous flow from NK progenitors towards fully mature NK cells (Figure 2B). Importantly, IL-2 was sufficient to cause NK cell maturation because higher numbers of pNK, iNK, and mNK were also observed in comparison to rIgG-treated control mice in the different developmental organs. However, a greater NK cell maturation occurred after CT. In conclusion, these data indicate that neutralization of TGF-β combined with IL-2 promoted NK cell recovery.

### 3.3. Early Recovery of NK Cells Co-expressing the Inhibitory Receptors NKG2A and Ly49G2 is Observed after NK Cell Stimulation during HSCT

**Figure 3:**
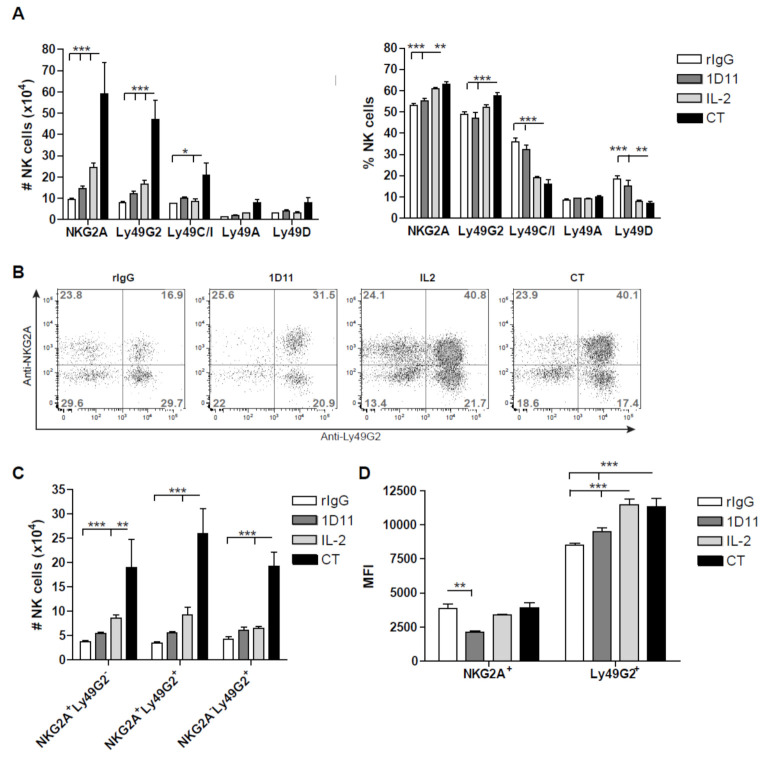
Preferential reconstitution and expansion of mature NK cells expressing Ly49G2 and NKG2A inhibitory receptors. Splenocytes were stained for different NK cell activating and inhibitory receptors at day 14 post-HSCT. (**A**) Total number and percentage of NK cells single-positive for NKG2A, Ly49G2, Ly49C/I, Ly49A and Ly49D suggest a higher presence of NKG2A^+^ and Ly49G2^+^ NK cell subsets after CT treatment. (**B**) Ly49G2 and NKG2A distribution is shown for gated NK cells (CD45.1^+^CD3^−^NK1.1^+^). (**C**) Total number of NK cells expressing Ly49G2 and NKG2A is shown. (**D**) Ly49G2 and NKG2A MFI is shown. Data are representative of two independent experiments with 3 mice per group (mean ± SEM). One-Way ANOVA was used to assess significance (* *p* < 0.05, ** *p* < 0.01, *** *p* < 0.001).

A preferential reconstitution and expansion of NK cells expressing the inhibitory receptor Ly49G2 after HSCT, viral infection or IL-2 stimulation has been previously reported by our group [29]. Analysis of multiple inhibitory and activating receptors at early time point post-HSCT confirmed our previous study but also demonstrated a further expansion of Ly49G2^+^ NK cell subset after NK cell stimulation which was significantly higher after CT treatment (Figure 3A). However, another inhibitory receptor, NKG2A, was also present in the vast majority of NK cells. Indeed, excluding the activating receptor NKG2D, which was expressed in approximately 90% of the NK cells (data not shown), NKG2A and Ly49G2 were the most prevalent NK cell receptors early after HSCT and particularly after CT treatment which was accompanied by a reduction of Ly49C/I^+^ and Ly49D^+^ NK cells upon activation (Figure 3A). Because Ly49G2^+^ NK cell expansion has been associated with activation in several NK stimulatory environments [29] and NKG2A^+^ NK cells are the first in recovering post-HSCT [37], we next investigated the distribution of both inhibitory receptors. Interestingly, the percentage of NKG2A^+^Ly49G2^+^ NK cells were significantly higher compared to NKG2A^−^ Ly49G2^+^, NKG2A^+^ Ly49G2^−^ and NKG2A^−^ Ly49G2^−^ NK cells after NK stimulation which also translated into greater numbers (Figure 3B,C). As previously reported [29], an increase of Ly49G2 median fluorescence intensity (MFI) was observed after CT and IL-2 treatment, but no differences were found for NKG2A (Figure 3D). This particular expansion of double-positive NKG2A Ly49G2 NK cell subset was also observed after IL-2 and CT treatment in resting mice. These data indicate that the acquisition of both Ly49G2 and NKG2A early after HSCT might represent an early activation phenotype, as was previously suggested for Ly49G2 [29].

### 3.4. CT Improves Reconstitution of Myeloid-derived Cells after HSCT

**Figure 4:**
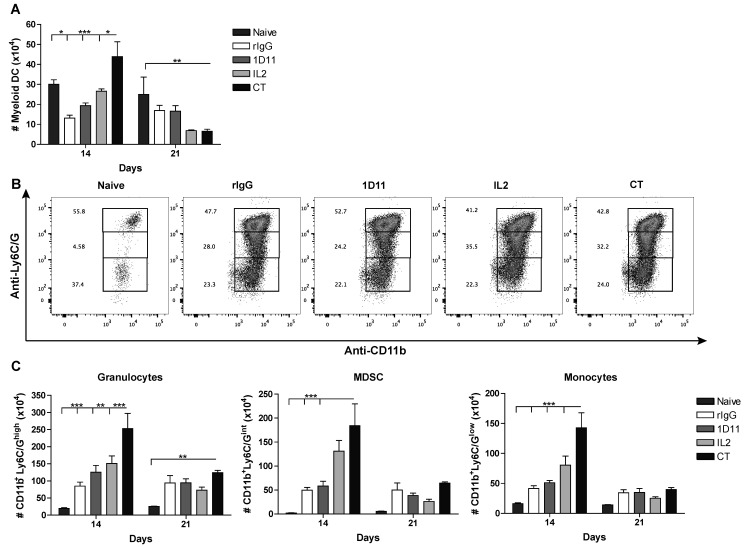
The reconstitution of myeloid-derived cells is faster and greater following CT therapy. Splenocytes were analyzed for myeloid-derived cells 24 h and 7 days after end of treatment (day 14 and 21 post-HSCT) by flow cytometry. (**A**) Total number of myeloid dendritic cells (CD45.1^+^CD3^−^CD19^−^CD11c^+^CD11b^+^) is shown. (**B**,**C**) Representative dot-plots of granulocytes (CD11b^+^Ly6C/G^high^), myeloid-derived suppressor cells (CD11b^+^Ly6C/G^int^) and monocytes (CD11b^+^Ly6C/G^low^) for days 14 post-HSCT is show. Cells were previously gated on CD45.1^+^CD3^−^CD19^−^CD11c^−^CD11b^+^ (**C**) Total number of granulocytes, myeloid-derived suppressor cells (MDSC) and monocytes for days 14 and 21 post-HSCT is shown. Data is representative of two independent experiments with 3 mice per group (mean ± SEM). One-Way ANOVA was used to assess significance (* *p* < 0.05, ** *p* < 0.01, *** *p* < 0.001).

Several studies have already demonstrated a role of TGF-β in the proliferation of HSC [38,39]. Indeed, TGF-β can inhibit the ex vivo formation of granulocyte-macrophage colonies derived from CD34^+^ cells [39]. It has also been suggested that this suppressive role is significantly dependent on the overall cytokine milieu and thus the balance between inflammatory and suppressive cytokines can determine the proliferative capabilities of HSC [25]. Because our immunotherapy strategy often leads to a more inflammatory environment, we expect that CT results in better reconstitution of myeloid-derived cells. We observed that the number of myeloid-derived dendritic cells (CD3^−^19^−^CD1c^+^CD11b^+^) was significantly higher in mice that received CT compared to control (rIgG) or IL-2-treated mice (Figure 4A). Additionally, a significant expansion of monocytes (CD3^−^CD19^−^CD11c^−^CD11b^+^Ly6C/G^low^), myeloid-derived suppressor cells (CD3^-^CD19^-^CD11c^-^CD11b^+^Ly6C/G^int^), and granulocytes (CD3^−^CD19^−^CD11c^−^CD11b^+^Ly6C/G^high^) was also observed (Figure 4B,C). Importantly, the level of myeloid-derived cells right after the end of immunotherapy was superior to the levels observed in naive resting mice, which could translate into a stronger protection against opportunistic infections. In correlation with the short half-life of both IL-2 and anti-TGF-β mAb 1D11, as well as the results obtained from the lymphocytic compartment, by day 21 post-HSCT there is an overall reduction of myeloid-derived cells. However, the levels of myeloid-derived cells still remain higher than the ones observed for naive resting mice. These results confirm the importance of the cytokine milieu in the development of myeloid cells and provide evidence of a faster and augmented reconstitution of myeloid-derived cells following IL-2 and anti-TGF-β immunotherapy.

### 3.5. Influence of CT on the NK cell Functional Properties

**Figure 5:**
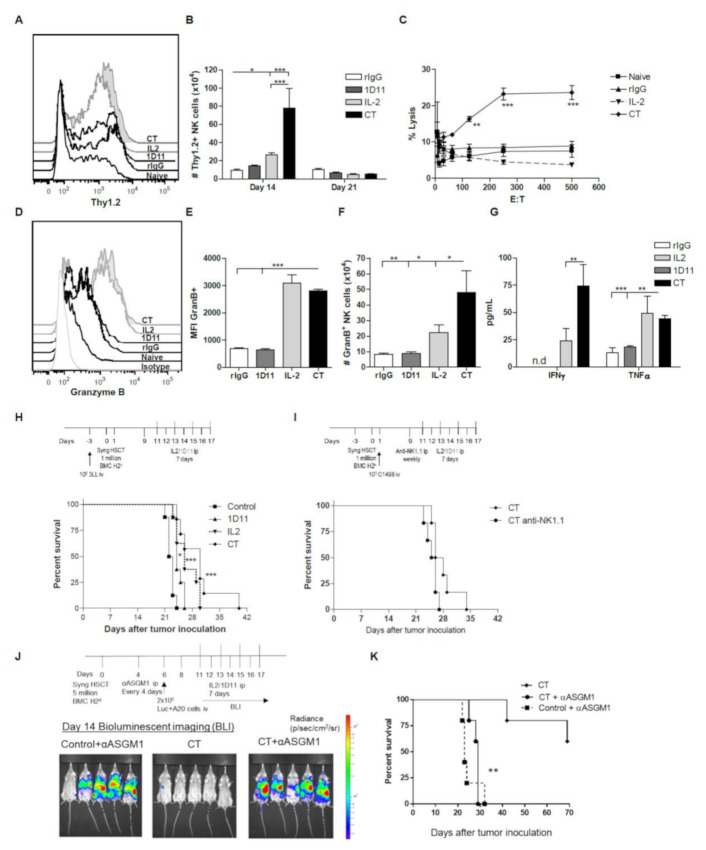
Impact of IL-2 and anti-TGF-β combinatorial therapy on NK cell function after HSCT. (**A**) Representative histograms are shown for Thy1.2 on gated NK cells (CD45.1^+^CD3^−^NK1.1^+^Thy1.2^+^). (**B**) The total number of Thy1.2^+^ NK cells is shown. (**C**) The total percentage of tumor lysis is shown for different effector: target (E:T) ratios on day 14 post-HSCT. (**D**–**F**) Representative histograms (**D**), median fluorescence intensity (MFI) (E), and total number of Granzyme B^+^ (GranB) NK cells (**F**) is shown for gated NK cells (CD45.1^+^CD3^−^NK1.1^+^) on day 14 post-HSCT. (**G**) Level of IFN-γ and TNF-α detected in the serum at day 14 post-HSCT. Data are representative of two independent experiments with three mice per group (mean ± SEM). C57BL/6 mice received 10^5^ tumor cells by iv injection four days prior to HSCT (3LL) or one day after HSCT (C1498). PBS/rIgG, anti-TGF-β (1D11) and/or IL-2 treatment started on day 11 after HSCT and lasted a week as stated previously. Some groups received ip injections of NK1.1 to deplete NK cells. (**H**,**I**) The percentage of survival is shown for 3LL (**H**) or C1498 (**I**) tumor-bearing mice. (**J**) Schema and bioluminescent images of A20 studies in BALB/c mice (images from day 14 post tumor inoculation). (**K**) Survival of mice challenged with A20 post-HSCT and treated with IL-2/TGF-β and indicated groups depleted of NK cells by anti-ASGM1. Data represents an experiment with five-eight mice per group. Statistical analysis was performed using Two-Way ANOVA, One-Way ANOVA or log-rank test when appropriate (* *p* < 0.05, ** *p* < 0.01, *** *p* < 0.001).

Notably, both IL-2 and CT treatment led to an increase in the murine activation marker Thy1.2 on the majority of NK cells compared to control treated groups (Figure 5A, Figure S3A), suggesting a higher activation status. However, due to the greater expansion of NK cells after CT treatment, the total number of Thy1.2^+^ NK cells was significantly higher than the IL-2 monotherapy group (Figure 5B). To demonstrate a superior functional capacity of CT-treated NK cells, splenocytes were co-cultured with the NK cell sensitive tumor cell line YAC-1, and the tumor lysis was evaluated in a standard 4 h Cr-release assay. We observed that a significantly higher percentage of lysis occurred in the group of mice receiving CT compared to IL-2 monotherapy (Figure 5C). In correlation with these results, heightened expression and absolute numbers of Granzyme B-positive (GranB) NK cells were also detected (Figure 5D–F, Figure S3B). Furthermore, elevated levels of IFN-γ in the serum of CT treated mice were observed (Figure 5G). These data indicate that NK cells from CT-treated mice display heightened function when compared to controls after HSCT. We then assessed effects on protection from tumor relapse. Mice were given lung carcinoma (3LL) iv and after four days were given congenic HSCT with or without CT or each agent given individually. The results showed that only the CT group demonstrated significant increases in survival (Figure 5H) demonstrating the accelerated NK cell recovery and activity could result in increased anti-tumor efficacy of HSCT. Depletion of NK cells by anti-NK1.1 administration following challenge with C1498 cells post-HSCT resulted in slightly more rapid mortality highlighting the protective role of NK cells in this leukemia model (Figure 5I). Using another strain (BALB/c) and tumor model (A20 lymphoma), depletion of NK cells with anti-asialo GM1 (anti-ASGM1) prior to CT treatment demonstrated that CT anti-tumor efficacy was NK cell dependent given the total loss in protection as shown by bioluminescent imaging and survival (Figure 5J,K). Overall, these results demonstrate the therapeutic potential of IL-2 and anti-TGF-β to meaningfully accelerate NK cell reconstitution and function without treatment-related toxicities resulting in greater protection from cancer relapse.

## 4. Discussion

Despite the recent impressive progress in cancer immunotherapy, significant hurdles remain. With autologous HSCT, immune deficiency preceding immune reconstitution represents a hurdle, limiting efficacy. Improved NK cell recovery following HSCT can potentially result in protection from cancer relapse and opportunistic infection [40]. Additionally, in allogeneic HSCT settings, the early recovery of NK cells could also minimize or even reduce the induction of GvHD, given the described role of NK cells in the control of alloreactive T cell responses responsible for the GvHD [41]. Further stimulation of NK cells through exogenous cytokine administration can be a plausible approach but the poor pharmacokinetics associated with systemic cytokine administration and toxicities result in limited efficacy. Indeed, high and frequent doses of IL-2 are required in order to obtain a biological significant expansion of NK cells but toxic side effects also occur [28], which can be amplified in patients that have gone through myeloablative conditioning for HSCT. Therefore, other approaches are needed to enhance NK cell reconstitution. Because administration of systemic IL-2 and neutralization of TGF-β or Treg depletion through anti-CD25 was shown to augment tumor survival in a CD8 T cell and/or NK cell dependent manner [13,27,42], the use of this immunotherapeutic regimen may also result in similar effects following HSCT. While the in vivo anti-tumor effects may not appear particularly striking, it is important to take into consideration that these were due to de novo generated NK cells in the immune-ablated recipient and immediately following lethal TBI and HSCT, when there is a tremendous skewing for myeloid and not lymphoid lineages.

Studies performed by other groups have illustrated the blockade of TGF-β as a possible therapeutic strategy [15,16,17,23,43]. The mechanism which augmented antitumor responses seems to be associated with the ability of anti-TGF-β to prevent tumor-sensitized Tregs [21]. There are reports that suggest a relative resistance of Tregs to radiation [44], but TGF-β can also be produced by many other cells such as stromal cells including fibroblast and endothelial cells [45]. Nevertheless, the lack of immunosuppression lead to an increase in other immunocompetent cell types, such as CD8, CD4 or NK cells. Consistent with these studies, we also showed that anti-TGF-β, in combination with IL-2, has a positive effect in NK cell expansion and function. This effect is likely due to the blockade of the immunosuppressive effect of Tregs as well, because this population underwent expansion following treatment with low dose of IL-2. This effect can be explained by the constitutive expression of the high affinity IL-2Rα (CD25) on Tregs that makes them more susceptible to IL-2 under conditions of low quantity, competing with NK and CD8 T cells, which express the IL-2Rcγ that displays lower affinity for IL-2 [12]. Moreover, we were also able to observe a positive impact in the precursor and immature NK cell progenitor compartments that suggests an accelerated NK cell development after HSCT. Unfortunately, in our model, the expansion of NK cells quickly reverted to baseline due to the short circulatory half-life of IL-2 (5 min) and anti-TGF-β 1D11 mAb (34 h) [31,32]. This effect was also observed after administration of this regimen in resting mice and correlates with the "cytokine addiction" effect already described that results in the loss of the NK cell population after the cessation of IL-2 treatment [33]. These results indicate that prolonged treatments could be needed given the strong homeostatic control pathways in place regarding NK cell numbers and activity. Some strategies that have been utilized to modulate the release of immunotherapeutic drugs include packing the drugs into nanoparticles [27]. Protein engineering approaches to reduce toxicity and to increase the half-life of cytokines have been also utilized, such is the case for the antibody-IL-2 immune complex [46]. However an NK cell exhaustion phenotype, characterized by impaired NK cell function, has been observed after prolonged stimulation [47,48,49,50] and therefore careful analysis of the cytokine stimulation regimen should be done.

Interestingly, after NK stimulation we observed the preferential expansion of NKG2D^+^ and NKG2A^+^Ly49G2^+^ NK cell subsets. NKG2D is an activating receptor and its expression has been correlated with better NK function. In allogeneic HSCT settings, it has been previously observed a reduction of GvHD due to the control of alloreactive T cells by NK cells in a NKG2D-dependent manner [41]. Given the higher expression of NKG2D after CT treatment in our syngeneic HSCT model, we could speculate that this regimen could also result in a stronger GvHD protection suggesting another clinical application although the inhibition of TGF-β may likely exacerbate GVHD.

NK development was augmented at both progenitor and mature cell levels and the inhibitory receptor phenotype of the reconstituting NK cells were of interest. In contrast to NKG2D, NKG2A and Ly49G2 are both inhibitory receptors [51]. Ly49G2 is an inhibitory receptor that binds to H2^d^ haplotype [52] and therefore its expression is associated with licensing in H2^d^ strains [53,54]. We demonstrated that the Ly49G2^+^ NK cell subset is preferentially expanded early after HSCT, cytokine stimulation, *Listeria monocytogenes,* and mouse cytomegalovirus (MCMV) infection independently of MHC class-I expression [29]. This Ly49G2^+^ NK cell subset was characterized by higher Ly49G2 expression measured by MFI and higher percentage of the activation marker Thy1.2. However, after IL-2 stimulation and despite the preferential Ly49G2^+^ NK expansion, the expression of Ly49G2 does not correlate with better NK function because sorted Ly49G2^+^Ly49C/I^-^ and Ly49G2^-^Ly49C/I^+^ are able to eliminate tumor cells at comparable levels suggesting that there was no an advantage of having a particular subset after strong NK activation [8]. NKG2A^+^ NK cells have also been shown to reconstitute early post-HSCT [55,56] and their presence has been associated with both impaired and improved NK function after HSCT [56,57,58]. For example, NKG2A^+^ NK cells showed the strongest response against K562 in an HLA-matched sibling HSCT study [57], whereas the co-expression of NKG2A and CD56^bright^ was correlated with an immature NK cell phenotype in HLA-matched HSCT [37,56]. Our findings demonstrated that the administration of activating signals (IL-2) in combination with the inhibition of suppressor signals (anti-TGF-β) accelerated the NK reconstitution at early time-points post-HSCT followed by robust NK cell expansion; which is characterized by better functional capabilities and Ly49G2 and NKG2A co-expression. Additionally, the development of myeloid-derived cells, as expected, was positively impacted by the combination IL-2 and anti-TGF-β regimen. The impact on NK cells could translate into beneficial anti-tumor and/or anti-viral effects limiting the rate of opportunistic infections and cancer relapse that follows shortly after HSCT. The stronger reconstitution of myeloid-derived cells could also account for improved responses against infection as has been shown with lethal challenge to fungal and bacterial infections [59]. One cannot rule out though that, in cancer, this increase in myeloid lineage development could also result in increased myeloid-derived suppressor cells (MDSC) which can be dominant in some cancers as an immune evasion pathway suggesting that more in-depth understanding of the myeloid effects in HSCT need to be delineated.

Alternatively, there are pre-clinical and clinical evidence for the promising synergistic potential of combining immunotherapy with chemotherapy that results in an improvement of the anti-tumor response in multiple cancers, such as breast cancer, metastatic melanoma, metastatic colorectal cancer, acute myeloid leukemia or renal cell carcinoma [60,61,62,63,64]. Similarly, the inhibition of the TGF-β signaling has shown to increase the chemotherapeutic effect in many types of cancers as well [65,66,67,68]. Taking into consideration these studies and our results, one could speculate that the IL-2/TGF-β blockade immunotherapy could also augment the chemotherapy-based anti-tumor responses outside of use in HSCT.

## 5. Conclusions

Our study thus demonstrates that combinatorial therapies, and particularly those that affect positive and negative regulators, may be more successful than single therapies and that accelerated NK cell reconstitution after HSCT can be a plausible alternative to adoptive transfer of NK cells to promote anti-tumor effects.

## Supplementary Materials

The following are available online at https://www.mdpi.com/2072-6694/12/11/3189/s1, Figure S1: The additive effect of anti-TGF- β towards IL-2 therapy is attenuated at high doses of IL-2, Figure S2: Impact of the anti-TGF- β towards IL-2 therapy in the Treg compartment, Figure S3: Immunotherapy treatment with anti-TGF- β and/or IL-2 results on NK cell activation.

## References

[1] Blazar B.R., Hill G.R., Murphy W.J. (2020). Dissecting the biology of allogeneic HSCT to enhance the GvT effect whilst minimizing GvHD. Nat. Rev. Clin. Oncol..

[2] Simonetta F., Alvarez M., Negrin R.S. (2017). Natural Killer Cells in Graft-versus-Host-Disease after Allogeneic Hematopoietic Cell Transplantation. Front. Immunol..

[3] Pierini A., Alvarez M., Negrin R.S. (2016). NK Cell and CD4+FoxP3+ Regulatory T Cell Based Therapies for Hematopoietic Stem Cell Engraftment. Stem Cells Int..

[4] Burns L.J., Weisdorf D.J., DeFor T.E., Vesole D.H., Repka T.L., Blazar B.R., Burger S.R., Panoskaltsis-Mortari A., Keever-Taylor C.A., Zhang M.J. (2003). IL-2-based immunotherapy after autologous transplantation for lymphoma and breast cancer induces immune activation and cytokine release: A phase I/II trial. Bone Marrow Transplant..

[5] Sutlu T., Alici E. (2009). Natural killer cell-based immunotherapy in cancer: Current insights and future prospects. J. Intern. Med..

[6] Elliott J.M., Yokoyama W.M. (2011). Unifying concepts of MHC-dependent natural killer cell education. Trends Immunol..

[7] Colucci F., Caligiuri M.A., Di Santo J.P. (2003). What does it take to make a natural killer?. Nat. Rev. Immunol..

[8] Alvarez M., Sun K., Murphy W.J. (2016). Mouse host unlicensed NK cells promote donor allogeneic bone marrow engraftment. Blood.

[9] Di Santo J.P. (2009). A defining factor for natural killer cell development. Nat. Immunol..

[10] Cooper M.A., Bush J.E., Fehniger T.A., VanDeusen J.B., Waite R.E., Liu Y., Aguila H.L., Caligiuri M.A. (2002). In vivo evidence for a dependence on interleukin 15 for survival of natural killer cells. Blood.

[11] Alvarez M., Ochoa M.C., Minute L., Melero I., Berraondo P. (2020). Rapid isolation and enrichment of mouse NK cells for experimental purposes. Methods Enzymol..

[12] Fehniger T.A., Cooper M.A., Caligiuri M.A. (2002). Interleukin-2 and interleukin-15: Immunotherapy for cancer. Cytokine Growth Factor Rev..

[13] Hallett W.H., Ames E., Alvarez M., Barao I., Taylor P.A., Blazar B.R., Murphy W.J. (2008). Combination therapy using IL-2 and anti-CD25 results in augmented natural killer cell-mediated antitumor responses. Biol. Blood Marrow Transpl..

[14] Boyman O., Surh C.D., Sprent J. (2006). Potential use of IL-2/anti-IL-2 antibody immune complexes for the treatment of cancer and autoimmune disease. Expert Opin. Biol. Ther..

[15] Li M.O., Wan Y.Y., Sanjabi S., Robertson A.K., Flavell R.A. (2006). Transforming growth factor-beta regulation of immune responses. Annu. Rev. Immunol..

[16] Ksendzovsky A., Feinstein D., Zengou R., Sharp A., Polak P., Lichtor T., Glick R.P. (2009). Investigation of immunosuppressive mechanisms in a mouse glioma model. J. Neuro-Oncol..

[17] Penafuerte C., Galipeau J. (2008). TGF beta secreted by B16 melanoma antagonizes cancer gene immunotherapy bystander effect. Cancer Immunol. Immunother..

[18] Rook A.H., Kehrl J.H., Wakefield L.M., Roberts A.B., Sporn M.B., Burlington D.B., Lane H.C., Fauci A.S. (1986). Effects of transforming growth factor beta on the functions of natural killer cells: Depressed cytolytic activity and blunting of interferon responsiveness. J. Immunol..

[19] Bellone G., Aste-Amezaga M., Trinchieri G., Rodeck U. (1995). Regulation of NK cell functions by TGF-beta 1. J. Immunol..

[20] Flavell R.A., Sanjabi S., Wrzesinski S.H., Licona-Limon P. (2010). The polarization of immune cells in the tumour environment by TGF-beta. Nat. Rev..

[21] Petrausch U., Jensen S.M., Twitty C., Poehlein C.H., Haley D.P., Walker E.B., Fox B.A. (2009). Disruption of TGF-beta signaling prevents the generation of tumor-sensitized regulatory T cells and facilitates therapeutic antitumor immunity. J. Immunol..

[22] Nam J.S., Terabe M., Mamura M., Kang M.J., Chae H., Stuelten C., Kohn E., Tang B., Sabzevari H., Anver M.R. (2008). An anti-transforming growth factor beta antibody suppresses metastasis via cooperative effects on multiple cell compartments. Cancer Res..

[23] Perry K., Wong L., Liu V., Park I., Zhang Q., Rejen V., Huang X., Smith N.D., Jovanovic B., Lonning S. (2008). Treatment of transforming growth factor-beta-insensitive mouse Renca tumor by transforming growth factor-beta elimination. Urology.

[24] Crane C.A., Han S.J., Barry J.J., Ahn B.J., Lanier L.L., Parsa A.T. (2010). TGF-beta downregulates the activating receptor NKG2D on NK cells and CD8+ T cells in glioma patients. Neuro-Oncol..

[25] Shah A.H., Tabayoyong W.B., Kimm S.Y., Kim S.J., Van Parijs L., Lee C. (2002). Reconstitution of lethally irradiated adult mice with dominant negative TGF-beta type II receptor-transduced bone marrow leads to myeloid expansion and inflammatory disease. J. Immunol..

[26] Ueda R., Fujita M., Zhu X., Sasaki K., Kastenhuber E.R., Kohanbash G., McDonald H.A., Harper J., Lonning S., Okada H. (2009). Systemic inhibition of transforming growth factor-beta in glioma-bearing mice improves the therapeutic efficacy of glioma-associated antigen peptide vaccines. Clin. Cancer Res..

[27] Park J., Wrzesinski S.H., Stern E., Look M., Criscione J., Ragheb R., Jay S.M., Demento S.L., Agawu A., Licona Limon P. (2012). Combination delivery of TGF-beta inhibitor and IL-2 by nanoscale liposomal polymeric gels enhances tumour immunotherapy. Nat. Mater..

[28] Alvarez M., Bouchlaka M.N., Sckisel G.D., Sungur C.M., Chen M., Murphy W.J. (2014). Increased Antitumor Effects Using IL-2 with Anti-TGF-beta Reveals Competition between Mouse NK and CD8 T Cells. J. Immunol..

[29] Barao I., Alvarez M., Ames E., Orr M.T., Stefanski H.E., Blazar B.R., Lanier L.L., Anderson S.K., Redelman D., Murphy W.J. (2011). Mouse Ly49G2+ NK cells dominate early responses during both immune reconstitution and activation independently of MHC. Blood.

[30] Alvarez M., Sungur C.M., Ames E., Anderson S.K., Pomeroy C., Murphy W.J. (2013). Contrasting effects of anti-Ly49A due to MHC class I cis binding on NK cell-mediated allogeneic bone marrow cell resistance. J. Immunol..

[31] Melder R.J., Osborn B.L., Riccobene T., Kanakaraj P., Wei P., Chen G., Stolow D., Halpern W.G., Migone T.S., Wang Q. (2005). Pharmacokinetics and in vitro and in vivo anti-tumor response of an interleukin-2-human serum albumin fusion protein in mice. Cancer Immunol. Immunother..

[32] Ling H., Li X., Jha S., Wang W., Karetskaya L., Pratt B., Ledbetter S. (2003). Therapeutic role of TGF-beta-neutralizing antibody in mouse cyclosporin A nephropathy: Morphologic improvement associated with functional preservation. J. Am. Soc. Nephrol..

[33] Miller J.S. (2013). Therapeutic applications: Natural killer cells in the clinic. Hematology/the Education Program of the American Society of Hematology. Am. Soc. Hematol. Educ. Prog..

[34] Marcoe J.P., Lim J.R., Schaubert K.L., Fodil-Cornu N., Matka M., McCubbrey A.L., Farr A.R., Vidal S.M., Laouar Y. (2012). TGF-beta is responsible for NK cell immaturity during ontogeny and increased susceptibility to infection during mouse infancy. Nat. Immunol..

[35] Hayakawa Y., Smyth M.J. (2006). CD27 dissects mature NK cells into two subsets with distinct responsiveness and migratory capacity. J. Immunol..

[36] Watt S.V., Andrews D.M., Takeda K., Smyth M.J., Hayakawa Y. (2008). IFN-gamma-dependent recruitment of mature CD27(high) NK cells to lymph nodes primed by dendritic cells. J. Immunol..

[37] Nguyen S., Dhedin N., Vernant J.P., Kuentz M., Al Jijakli A., Rouas-Freiss N., Carosella E.D., Boudifa A., Debre P., Vieillard V. (2005). NK-cell reconstitution after haploidentical hematopoietic stem-cell transplantations: Immaturity of NK cells and inhibitory effect of NKG2A override GvL effect. Blood.

[38] Park S.M., Deering R.P., Lu Y., Tivnan P., Lianoglou S., Al-Shahrour F., Ebert B.L., Hacohen N., Leslie C., Daley G.Q. (2014). Musashi-2 controls cell fate, lineage bias, and TGF-beta signaling in HSCs. J. Exp. Med..

[39] Garbe A., Spyridonidis A., Mobest D., Schmoor C., Mertelsmann R., Henschler R. (1997). Transforming growth factor-beta 1 delays formation of granulocyte-macrophage colony-forming cells, but spares more primitive progenitors during ex vivo expansion of CD34+ haemopoietic progenitor cells. Br. J. Haematol..

[40] Ruggeri L., Capanni M., Urbani E., Perruccio K., Shlomchik W.D., Tosti A., Posati S., Rogaia D., Frassoni F., Aversa F. (2002). Effectiveness of donor natural killer cell alloreactivity in mismatched hematopoietic transplants. Science.

[41] Olson J.A., Leveson-Gower D.B., Gill S., Baker J., Beilhack A., Negrin R.S. (2010). NK cells mediate reduction of GVHD by inhibiting activated, alloreactive T cells while retaining GVT effects. Blood.

[42] Wojtowicz-Praga S., Verma U.N., Wakefield L., Esteban J.M., Hartmann D., Mazumder A. (1996). Modulation of B16 melanoma growth and metastasis by anti-transforming growth factor beta antibody and interleukin-2. J. Immunother..

[43] Qin W., Tian F., Wang F., Song B., Wang H., Zhang Q., Jovanovic B., Liang L., Guo Y., Smith N. (2008). Adoptive transfer of tumor-reactive transforming growth factor-beta-insensitive cytolytic T cells for treatment of established mouse Renca tumors. Urology.

[44] De Luca A., Fiorillo M., Peiris-Pages M., Ozsvari B., Smith D.L., Sanchez-Alvarez R., Martinez-Outschoorn U.E., Cappello A.R., Pezzi V., Lisanti M.P. (2015). Mitochondrial biogenesis is required for the anchorage-independent survival and propagation of stem-like cancer cells. Oncotarget.

[45] Carli C., Giroux M., Delisle J.S. (2012). Roles of transforming growth factor-beta in graft-versus-host and graft-versus-tumor effects. Biol. Blood Marrow Transpl..

[46] Letourneau S., van Leeuwen E.M., Krieg C., Martin C., Pantaleo G., Sprent J., Surh C.D., Boyman O. (2010). IL-2/anti-IL-2 antibody complexes show strong biological activity by avoiding interaction with IL-2 receptor alpha subunit CD25. Proc. Natl. Acad. Sci. USA.

[47] Alvarez M., Simonetta F., Baker J., Pierini A., Wenokur A.S., Morrison A.R., Murphy W.J., Negrin R.S. (2019). Regulation of murine NK cell exhaustion through the activation of the DNA damage repair pathway. JCI Insight.

[48] Alvarez M., Simonetta F., Baker J., Morrison A.R., Wenokur A.S., Pierini A., Berraondo P., Negrin R.S. (2020). Indirect Impact of PD-1/PD-L1 Blockade on a Murine Model of NK Cell Exhaustion. Front. Immunol..

[49] Elpek K.G., Rubinstein M.P., Bellemare-Pelletier A., Goldrath A.W., Turley S.J. (2010). Mature natural killer cells with phenotypic and functional alterations accumulate upon sustained stimulation with IL-15/IL-15Ralpha complexes. Proc. Natl. Acad. Sci. USA.

[50] Gill S., Vasey A.E., De Souza A., Baker J., Smith A.T., Kohrt H.E., Florek M., Gibbs K.D., Tate K., Ritchie D.S. (2012). Rapid development of exhaustion and down-regulation of eomesodermin limit the antitumor activity of adoptively transferred murine natural killer cells. Blood.

[51] Lanier L.L. (2005). NK cell recognition. Annu. Rev. Immunol..

[52] Hanke T., Takizawa H., McMahon C.W., Busch D.H., Pamer E.G., Miller J.D., Altman J.D., Liu Y., Cado D., Lemonnier F.A. (1999). Direct assessment of MHC class I binding by seven Ly49 inhibitory NK cell receptors. Immunity.

[53] Kim S., Poursine-Laurent J., Truscott S.M., Lybarger L., Song Y.J., Yang L., French A.R., Sunwoo J.B., Lemieux S., Hansen T.H. (2005). Licensing of natural killer cells by host major histocompatibility complex class I molecules. Nature.

[54] Sun K., Alvarez M., Ames E., Barao I., Chen M., Longo D.L., Redelman D., Murphy W.J. (2012). Mouse NK cell-mediated rejection of bone marrow allografts exhibits patterns consistent with Ly49 subset licensing. Blood.

[55] Miller J.S., McCullar V. (2001). Human natural killer cells with polyclonal lectin and immunoglobulinlike receptors develop from single hematopoietic stem cells with preferential expression of NKG2A and KIR2DL2/L3/S2. Blood.

[56] Dulphy N., Haas P., Busson M., Belhadj S., Peffault de Latour R., Robin M., Carmagnat M., Loiseau P., Tamouza R., Scieux C. (2008). An unusual CD56(bright) CD16(low) NK cell subset dominates the early posttransplant period following HLA-matched hematopoietic stem cell transplantation. J. Immunol..

[57] Bjorkstrom N.K., Riese P., Heuts F., Andersson S., Fauriat C., Ivarsson M.A., Bjorklund A.T., Flodstrom-Tullberg M., Michaelsson J., Rottenberg M.E. (2010). Expression patterns of NKG2A, KIR, and CD57 define a process of CD56dim NK-cell differentiation uncoupled from NK-cell education. Blood.

[58] Foley B., Cooley S., Verneris M.R., Pitt M., Curtsinger J., Luo X., Lopez-Verges S., Lanier L.L., Weisdorf D., Miller J.S. (2012). Cytomegalovirus reactivation after allogeneic transplantation promotes a lasting increase in educated NKG2C+ natural killer cells with potent function. Blood.

[59] BitMansour A., Burns S.M., Traver D., Akashi K., Contag C.H., Weissman I.L., Brown J.M. (2002). Myeloid progenitors protect against invasive aspergillosis and Pseudomonas aeruginosa infection following hematopoietic stem cell transplantation. Blood.

[60] Caraglia M., Correale P., Giannicola R., Staropoli N., Botta C., Pastina P., Nesci A., Caporlingua N., Francini E., Ridolfi L. (2019). GOLFIG Chemo-Immunotherapy in Metastatic Colorectal Cancer Patients. A Critical Review on a Long-Lasting Follow-Up. Front. Oncol..

[61] Eigentler T.K., Weide B., de Braud F., Spitaleri G., Romanini A., Pflugfelder A., Gonzalez-Iglesias R., Tasciotti A., Giovannoni L., Schwager K. (2011). A dose-escalation and signal-generating study of the immunocytokine L19-IL-2 in combination with dacarbazine for the therapy of patients with metastatic melanoma. Clin. Cancer Res..

[62] Johannsen M., Spitaleri G., Curigliano G., Roigas J., Weikert S., Kempkensteffen C., Roemer A., Kloeters C., Rogalla P., Pecher G. (2010). The tumour-targeting human L19-IL-2 immunocytokine: Preclinical safety studies, phase I clinical trial in patients with solid tumours and expansion into patients with advanced renal cell carcinoma. Eur. J. Cancer.

[63] Pretto F., Elia G., Castioni N., Neri D. (2014). Preclinical evaluation of IL-2-based immunocytokines supports their use in combination with dacarbazine, paclitaxel and TNF-based immunotherapy. Cancer Immunol. Immunother..

[64] Schliemann C., Gutbrodt K.L., Kerkhoff A., Pohlen M., Wiebe S., Silling G., Angenendt L., Kessler T., Mesters R.M., Giovannoni L. (2015). Targeting interleukin-2 to the bone marrow stroma for therapy of acute myeloid leukemia relapsing after allogeneic hematopoietic stem cell transplantation. Cancer Immunol. Res..

[65] Liu Q., Wu H., Li Y., Zhang R., Kleeff J., Zhang X., Cui M., Liu J., Li T., Gao J. (2020). Combined blockade of TGF-beta1 and GM-CSF improves chemotherapeutic effects for pancreatic cancer by modulating tumor microenvironment. Cancer Immunol. Immunother..

[66] Guillen Diaz-Maroto N., Sanz-Pamplona R., Berdiel-Acer M., Cimas F.J., Garcia E., Goncalves-Ribeiro S., Albert N., Garcia-Vicien G., Capella G., Moreno V. (2019). Noncanonical TGF-beta Pathway Relieves the Blockade of IL1beta/TGF-beta-Mediated Crosstalk between Tumor and Stroma: TGF-BR1 and TAK1 Inhibition in Colorectal Cancer. Clin. Cancer Res..

[67] Newsted D., Banerjee S., Watt K., Nersesian S., Truesdell P., Blazer L.L., Cardarelli L., Adams J.J., Sidhu S.S., Craig A.W. (2019). Blockade of TGF-beta signaling with novel synthetic antibodies limits immune exclusion and improves chemotherapy response in metastatic ovarian cancer models. Oncoimmunology.

[68] Farokhzad O.C., Cheng J., Teply B.A., Sherifi I., Jon S., Kantoff P.W., Richie J.P., Langer R. (2006). Targeted nanoparticle-aptamer bioconjugates for cancer chemotherapy in vivo. Proc. Natl. Acad. Sci. USA.

