# Supplementary material for: IL-2 and Anti-TGF-β Promote NK Cell Reconstitution and Anti-tumor Effects after Syngeneic Hematopoietic Stem Cell Transplantation
Source: Cancers (Basel). 2020 Oct 29;12(11):3189. doi: 10.3390/cancers12113189 (PMC7692743; doi:10.3390/cancers12113189)
Supplement: Supplementary file 1 [file cancers-12-03189-s001.zip › cancers-964045-supplementary.docx]

IL-2 and Anti-TGF-β Promote NK Cell Reconstitution and Anti-tumor Effects after Syngeneic Hematopoietic Stem Cell Transplantation

Maite Alvarez, Cordelia Dunai, Lam T. Khuat, Ethan G. Aguilar, Isabel Barao, and William J. Murphy


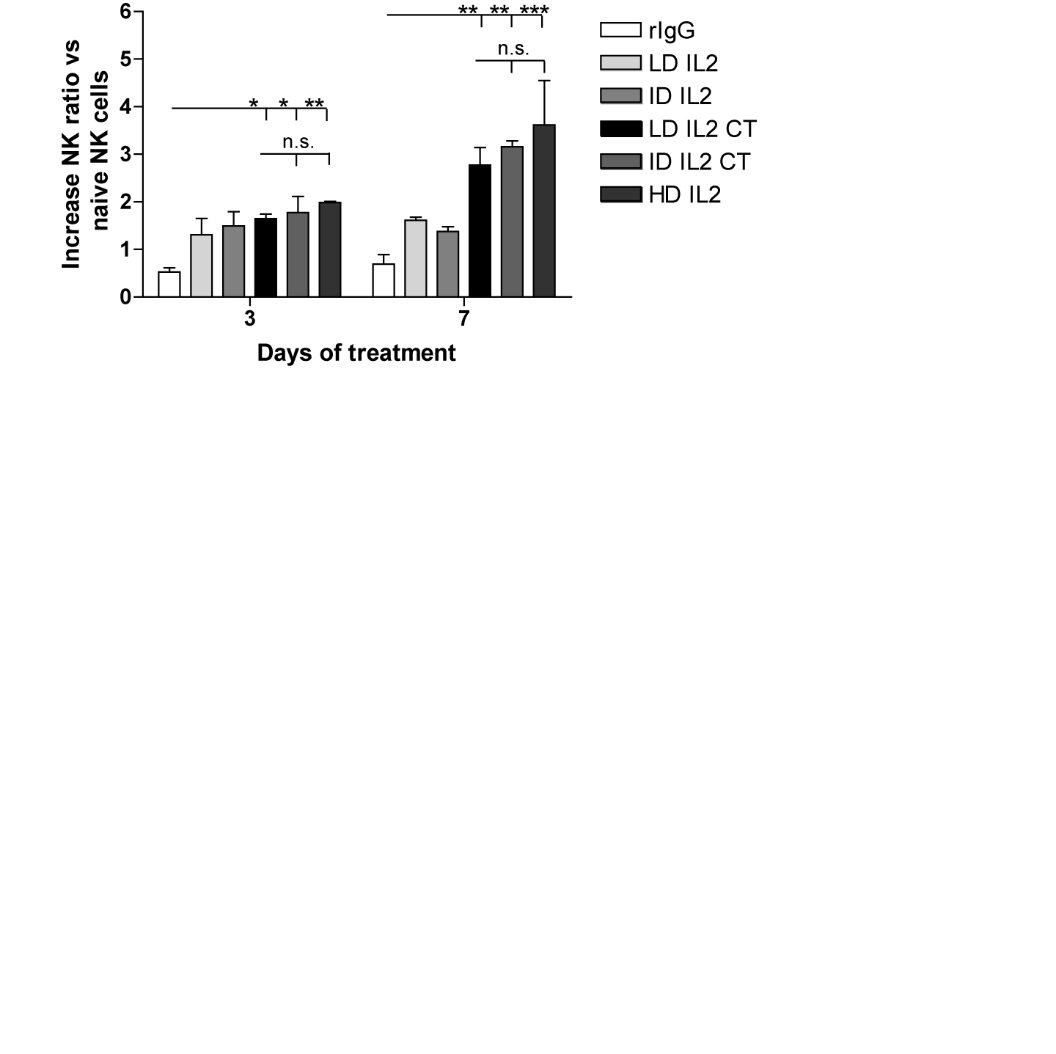


**Figure S1.** The additive effect of anti-TGF-β towards IL-2 therapy is attenuated at high doses of IL-2. C57BL/6 mice received congenic HSCT and were treated as previously described with low dose (LD: 2 × 10^5^ IU), intermediate dose (ID: 5 × 10^5^ IU) or high dose (HD: 10^6^ IU) of IL-2 +/- anti-TGFβ for three or seven days. Data shows the total number of NK cells (CD45^+^CD3^-^NK1.1^+^) after HSCT compared to naive NK cells. Data is representative of two experiments with three mice per group (mean ± SEM). One-Way ANOVA was used to assess significance (**p* < 0.05, ***p* < 0.01, ****p* < 0.001; n.s no significance).


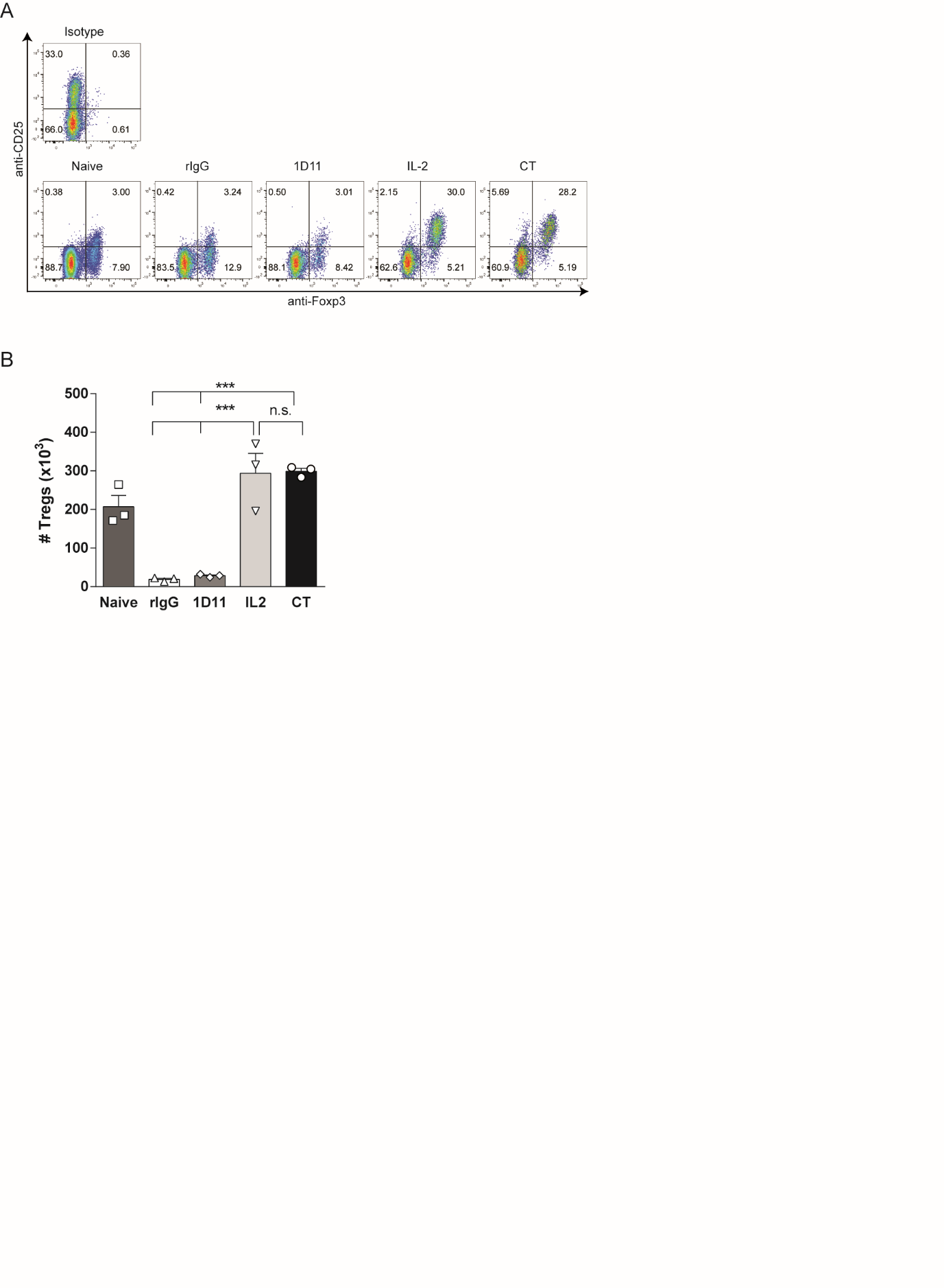


**Figure S2.** Impact of the anti-TGF-β towards IL-2 therapy in the Treg compartment. **(A)** Representative dot-plots of Foxp3 and CD25 expression on gated CD3^+^CD4^+^ T cells. **(B)** Total number of Tregs (CD3^+^CD4^+^Foxp3^+^CD25^+^) is shown 14 days post-HSCT. Data is representative of two experiments with 3 mice per group (mean ± SEM). One-Way ANOVA was used to assess significance (****p* < 0.001; n.s no significance).


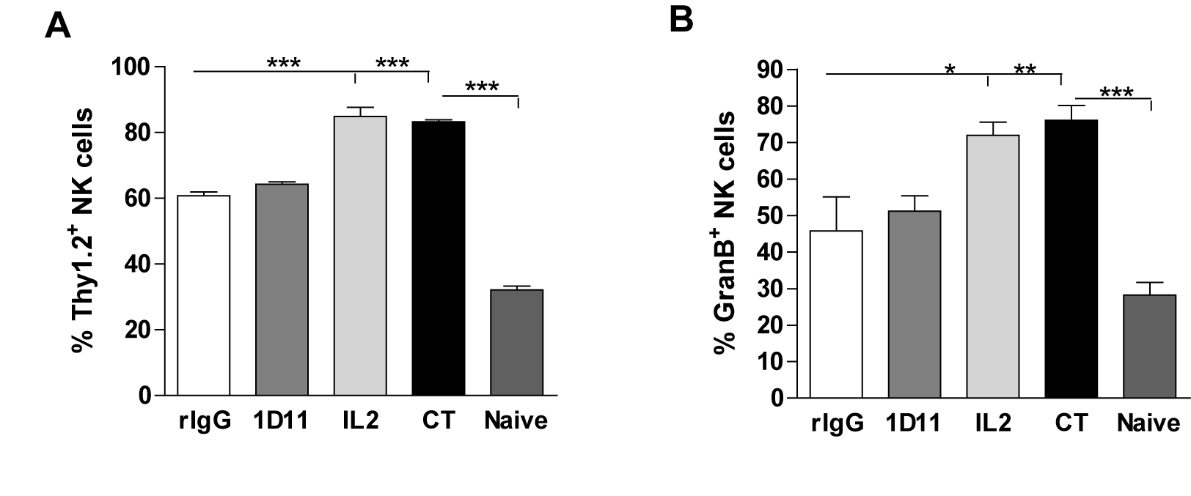


**Figure S3.** Immunotherapy treatment with anti-TGFβ and/or IL-2 results in NK cell activation. The treatment with IL-2 and CT increases the percentage of the activation marker Thy1.2 **(A)** and granzyme B **(B)** on gated NK cells (CD45^+^CD3^-^NK1.1^+^). Data are representative of two experiments with 3 mice per group (mean ± SEM). One-Way ANOVA was used to assess statistical significance (**p* < 0.05, ***p* < 0.01, ****p* < 0.001).
